# A type I combi-targeting approach for the design of molecules with enhanced potency against BRCA1/2 mutant- and O6-methylguanine-DNA methyltransferase (mgmt)- expressing tumour cells

**DOI:** 10.1186/s12885-017-3504-1

**Published:** 2017-08-11

**Authors:** Zhor Senhaji Mouhri, Elliot Goodfellow, Bertrand Jean-Claude

**Affiliations:** Cancer Drug Research Laboratory, Department of Medicine, Division of Medical Oncology, McGill University Health Center/Royal Victoria Hospital, 1001 Decarie boul, Montreal, QC H4A 3J1 Canada

**Keywords:** Chemoresistance, Temozolomide, MGMT, BRCA1/2 reactivation, PARP inhibitor, Combi-targeting, DNA repair, 1,2,3-methyltriazene

## Abstract

**Background:**

Mutations of the DNA repair proteins BRCA1/2 are synthetically lethal with the DNA repair enzyme poly(ADP-ribose) polymerase (PARP), which when inhibited, leads to cell death due to the absence of compensatory DNA repair mechanism. The potency of PARP inhibitors has now been clinically proven. However, disappointingly, acquired resistance mediated by the reactivation of wild type BRCA1/2 has been reported. In order to improve their efficacy, trials are ongoing to explore their combinations with temozolomide (TMZ). Here, in order to enhance potency in BRCA1/2-mutant cells, we report on the design of single molecules termed “combi-molecules” capable of not only inhibiting PARP but also damaging DNA like TMZ, which is known to induce a large number of DNA adducts. The majority of these lesions are processed through PARP-dependent base-excision repair machinery. Paradoxically, the least abundant lesion, the O6-methylguanine adduct is the most cytotoxic. Its repair by the O6-methylguanine DNA methyl transferase (MGMT) confers robust resistance to TMZ. Thus, we surmise that a combi-molecule designed to generate the same DNA adducts as TMZ, with an additional ability to block PARP, could induce BRCA1/2 mutant selective potency and a growth inhibitory profile independent of MGMT status.

**Methods:**

The hydrolysis of EG22 and its stabilized form ZSM02 was analyzed by HPLC and fluorescence spectroscopy. Growth inhibitory potency was determined by SRB assay. PARP inhibition was determined by an enzyme assay and DNA damage by the comet assay. Subcellular distribution was visualized by confocal microscopy.

**Results:**

Studies on EG22 showed that: (a) it inflicted anomalously higher levels of DNA damage than TMZ (b) it induced PARP inhibitory potency in the same range as ANI, a known PARP inhibitor (IC50 = 0.10 μM) (c) it showed strong potency in both BRCA1/2 wild type and mutated cells with 6-fold selectivity for the mutants and it was 65–303-fold more potent than TMZ and 4–63-fold than ANI alone and 3–47-fold than their corresponding equimolar combinations and (d) its potency was independent of MGMT expression.

**Conclusion:**

The results in toto suggest that a combi-molecular approach directed at blocking PARP and damaging DNA can lead to single molecules with selective and enhanced potency against BRCA1/2 mutant and with activity independent of MGMT, the major predictive biomarker for resistance to TMZ.

## Background

**Fig. 1:**
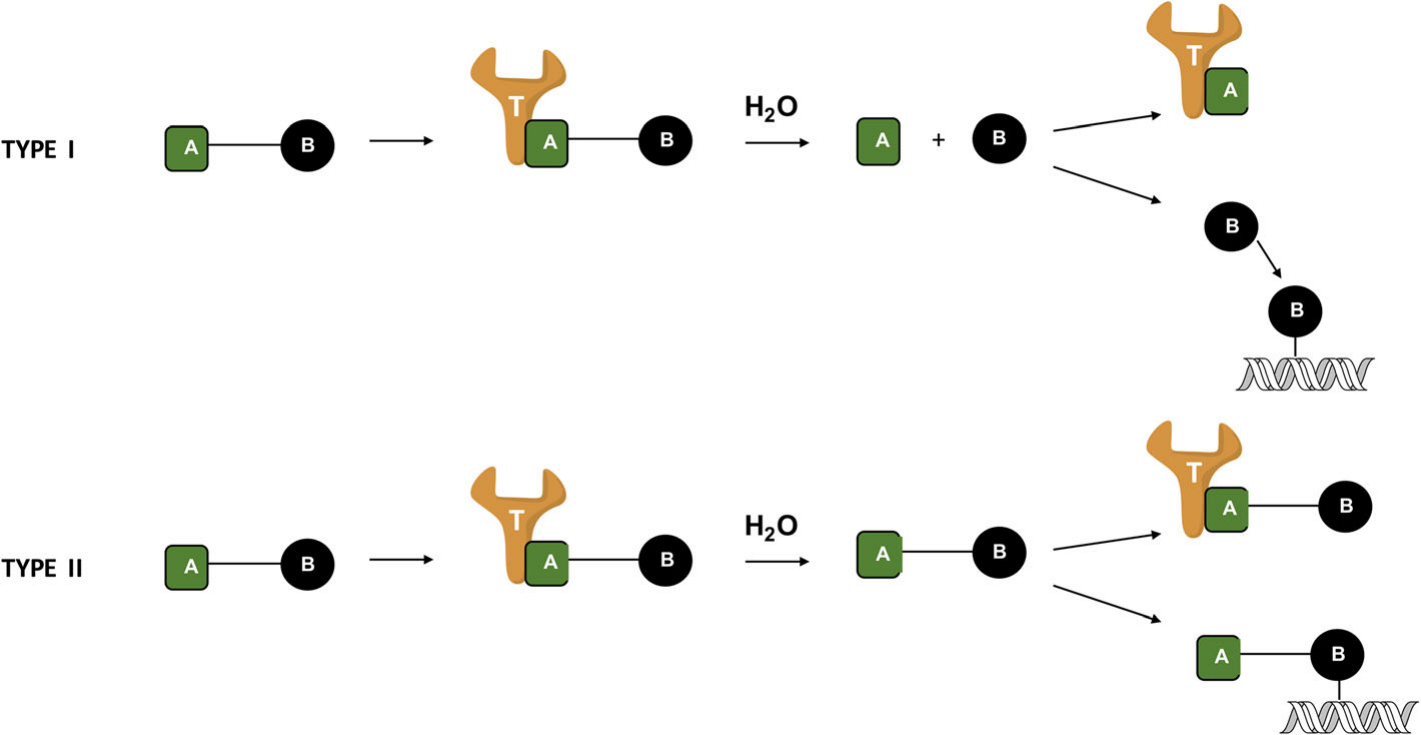
Schematic representation of type I and type II combi-molecules. Upon entering the cells, type I combi-molecules are able to bind and inhibit their target as intact molecules. In the cells, the molecule is hydrolyzed to release an inhibitor ‘A’ and a DNA damaging agent ‘B’. Type II combi-molecules enter the cells and are able to inhibit their target and damage DNA without hydrolysis. Inhibition of the target can synergize with the effects of the DNA damaging species

**Fig. 2:**
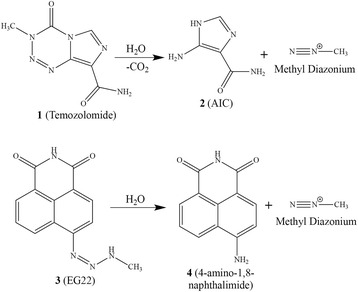
*top*: Hydrolysis of TMZ to generate the inactive AIC and the methyl diazonium species; *bottom*: Hydrolysis of EG22 to regenerate ANI, the naked PARP inhibitor, and the same methyl diazonium species as temozolomide

Over the past decade, a new strategy to target DNA repair deficiency has progressed to clinical trials: synthetic lethality. The concept of synthetic lethality applies to a situation where mutation of gene A or B alone does not affect the viability of a cell. However, mutation of both genes leads to cell death [1–4]. A typical case of synthetic lethality is that of cells expressing the mutant BRCA1 or 2. Loss of BRCA1/2 functions impair the DNA repair process. On the other hand, the base excision repair protein PARP is critical for compensating for the loss of BRCA1/2 by providing an alternative DNA repair function to the cells. Thus, concomitant loss of function of the BRCA1/2 genes and PARP induces significant genomic instability and this ultimately leads to cell death [1, 2, 4]. This situation is produced by using inhibitors to block PARP function in BRCA1/2 mutant cells. Thus, PARP inhibitors selectively kill tumour cells with disordered expression of BRCA1/2 (mutation or loss) [1, 4]. Olaparib, the first PARP inhibitor approved in the clinic has proven effective in the treatment of ovarian tumours characterized by BRCA1/2 mutations [5–7] and many other trials are ongoing to demonstrate the potency of other PARP inhibitors in BRCA1/2 tumours [8]. Disappointingly, clinical trials revealed that some patients become resistant to PARP inhibitors and this is believed to be due to genetic reversion that corrects the original BRCA1- or 2-inactivating mutation [9, 10]. Therefore, strategies to augment the potency of the approach in BRCA1/2 mutant cells are urgently needed. Here we surmised that a small molecule capable of not only blocking PARP, but also damaging DNA, would be a more effective agent against BCRA1/2 mutants than a PARP-specific inhibitor. The design of such a type of molecule was based upon a principle developed by our group termed: “the combi-targeting concept”, which, as outlined in Fig. 1, postulates that a small molecule AB kept small enough to be bound to its target T and capable of generating, upon hydrolysis, another inhibitor A of the same target + another bioactive molecule B (e.g a DNA damaging species), should induce greater potency than its single targeted counterpart. Importantly, we surmised that due to its targeted property, such a type of molecule could also be more potent than combinations of the two agents A (inhibitor) + B (DNA damaging species) or their corresponding analogues with identical mechanisms action [11, 12]. As depicted in Fig. 2, the molecules that requires hydrolytic cleavage to exert its activity is termed: “type I combi-molecules” as opposed to type II combi-molecules that do not require hydrolytic cleavage. Here we design a combi-molecule to inhibit PARP and to release a DNA damaging species (methyldiazonium), the same agents known to be responsible for the cytotoxicity of temozolomide (TMZ) [13, 14] (Fig. 2).

On the other hand, because of the sensitivity of BRCA1/2 mutant cells to DNA damaging agents, the most studied combinations designed to enhance the potency of PARP inhibitors involve alkylating agents like TMZ, a second generation alkyltriazene that is used in the treatment of glioblastoma and melanoma [15–17]. The hydrolysis of TMZ under physiological conditions leads to 5-aminoimidazole-4-carboxamide (AIC) and a methyldiazonium ion (Fig. 2) that reacts with DNA to create N3-methyladenine, N7-methylguanine, N7-methyladenine and O6-methylguanine adducts [18]. The clinical potency of TMZ is significantly affected by the expression O6-methylguanine DNA methyl transferase (MGMT) [14, 19], a DNA repair enzyme that removes the methyl group from guanine by transferring it to its own cystein residue [14, 20]. The other types of lesions induced by TMZ (e.g. N7-methylguanine and N3-methyladenine) are processed by the base excision repair machinery, in which PARP plays a central role. It has already been shown that in MGMT-proficient cells, PARP inhibition sensitized cells to TMZ [21–23] and this was believed to be due to the cytotoxic effects of unrepaired alkylated bases other than O6-methylguanine. Accordingly, given that the mechanism of cell-killing by the designed combi-molecule in BRCA1/2 depends on PARP inhibition, we also sought to determine whether the MGMT status of the cells would influence the potency of these dual PARP-DNA targeting combi-molecules.

To achieve synthetic lethality–directed combi-molecules, we exploited the chemistry of open-chain and cyclic 1,2,3-triazenes, which has led to the synthesis of the potent clinical alkylating agent TMZ. The hydrolysis of both open-chain or cyclic triazene ultimately leads to the formation of an aromatic amine and a DNA alkylating species [24]. Thus, we designed EG22 to contain a hydrolabile 1,2,3-triazene link that masks a PARP inhibitor, 4-amino-1,8-naphthalimide (ANI) and a methyldiazonium species (Fig. 2). Here we report on the synthesis and the dual targeting properties of EG22, the first open-chain and dual targeted PARP-DNA combi-molecule ever synthesized. Furthermore, since the hydrolysis of EG22 was rather fast under physiological conditions, we also report herein the synthesis and growth inhibitory profile of its acetylated form designed to delay its hydrolysis, thereby stabilizing it under physiological conditions.

## Methods

### Chemicals and reagents

ANI was purchased from AstaTech Inc. All the chemical reagents and solvents were purchased from Sigma Aldrich Canada.

### Chemistry

#### 6-(3-Methyltriaz-1-en-1-yl)-1H–benzo[de]isoquinoline-1,3(2H)-dione (3)

**Fig. 3:**
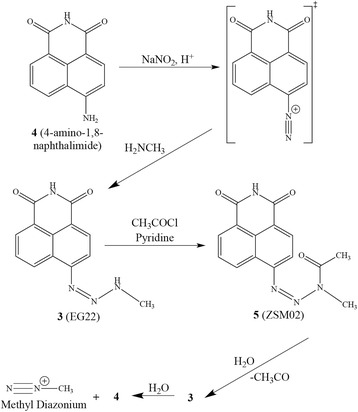
Synthesis of ZSM02 from the acetylation of EG22 and its hydrolysis under physiological conditions

EG22 (**3**) was synthesized as described in Fig. 3. The synthesis of its ^15^N and ^13^C–labeled form for purpose of characterization was reported elsewhere [25]. Briefly, 4-amino-1,8-naphthalimide (ANI, **4**) (50.0 mg, 1 eq, 0.236 mmol) was dissolved in concentrated trifluoroacetic acid (5 mL) and the resulting solution cooled to -5 °C for 15 min. An aqueous solution (1 mL) of sodium nitrite (32.5 mg, 2 eq, 0.472 mmol) was subsequently added dropwise and the solution kept at −5 °C for 15 min, thereafter, methylamine (40% *v*/v) (0.122 mL, 6 eq, 1.41 mmol) was added dropwise. The solution was subsequently neutralized with a saturated solution of sodium bicarbonate and the precipitate that formed collected and dried overnight in vacuo to give **2** as a brown powder. ^1^H NMR (400 MHz, DMSO-*d*_*6*_) δ ppm 11.59 (s, 1H, NH), 11.42 (q, 1H, J = 4.0 Hz, NHCH_3_), 8.98 (dd, 1H, J = 8.5 Hz, 1.3 Hz, ArH), 8.47 (dd, 1H, J = 7.2 Hz, 1.2 Hz, ArH), 8.40 (d, 1H, J = 8.1 Hz, ArH), 7.84 (t, 1H, J = 7.9 Hz, ArH), 7.69 (d, 1H, J = 8.1 Hz, ArH), 3.26 (d, 3H, J = 3.9 Hz, CH_3_NH). ESI m/z 253.0732 (MH^−^).

#### 6-(3-Acetyl-3-methyltriaz-1-en-1-yl)-1H–benzo[de]isoquinoline-1,3(2H)-dione (5):

The acetylated compound ZSM02 (**3**) was synthesized as depicted in Fig. 3 and methods for the preparation of its isotopically labeled form for purpose of characterization was reported elsewhere [25]. Briefly, anhydrous pyridine (3 mL) was flash frozen in liquid nitrogen. Acetic anhydride (0.186 mL, 10 eq, 1.97 mmol) was introduced all at once thereafter. The triazene (**2**) in Fig. 3 (50.0 mg, 1 eq, 0.197 mmol) was added as a powder. The reaction was allowed to reach a temperature of -5 °C for 30 min and then reached room temperature slowly for 2 h. Once the reaction was complete, the pyridine was removed using toluene to create an azeotrope and the resulting solid collected, dried and purified by preparative HPLC (acetonitrile/water: 50/50). ^1^H NMR (400 MHz, DMSO-*d*_*6*_) δ ppm 11.83 (s, 1H, NH), 8.96 (dd, 1H, J = 8.5 Hz, 1.2 Hz, ArH), 8.54 (dd, 1H, J = 7.3 Hz, 1.2 Hz, ArH), 8.51 (d, 1H, J = 7.9 Hz, ArH), 7.96 (t, 1H, J = 7.9 Hz, ArH), 7.94 (d, 1H, J = 8.0 Hz, ArH), 3.54 (s, 3H, CH_3_N), 2.60 (s, 3H, CH_3_CO). ^13^C NMR (400 MHz, DMSO-*d*_*6*_) δ ppm 172.96, 163.71, 148.35, (2C) 130.56, 130.53, 129.78, 129.69, 127.72, 127.57, 122.74, 122.18, 114.33, 28.17 and 22.04. ESI m/z 297 (MH^−^).

### Cell culture

VC8, VC8-BRCA, and V79 Chinese Hamster Lung cells were generously provided by Dr. Bernd Kaina (Institute of Toxicology, Mainz, Germany). T98 glioblastoma cell lines were kindly given by Dr. Siham Sabri (the Research Institute of the McGill University Health Centre, Montreal, Canada). A549 (ATCC® CCL-185™), DU145 (ATCC® HTB-85™), and A427 (ATCC® HTB-53™), was purchased from ATCC. A427-MGMT was obtained by stable transfection of A427 with MGMT viral vector in our lab [26]. All cell lines were maintained in in DMEM media from Wisent Bio Products. Media preparation was supplemented with 10% Fetal Bovine Serum (FBS), 12 mL HEPES, 5 mL L-glutamine, 500 μL of gentamicin sulfate, 250 μL of fungisome, and 170 μL of ciprofloxacin. All the bio-products used in the preparation of the media were purchased from Wisent Inc. The cells were grown in Thermo Scientific™ BioLite Cell Culture Treated Flasks cell cultured treated polystyrene flasks, which are placed in an incubator with a stable temperature of 37 °C and CO_2_ level of 5%. The media of each flask was changed when necessary and cell passaging was performed at 85 and 95% confluence.

### In vitro growth inhibition assay

Growth inhibitory potency was evaluated using the SRB assay [27]. Briefly, cells were plated in 96-well in triplicate and treated with drugs (0.078 μM to 100 μM) 24 h after seeding. Following drug treatment, the cells were fixed using 50 μl of cold TCA (50%) for 1 h at 4 °C, washed five times with tap water, and stained for 30 min at room temperature with SRB (0.4%) in acetic acid (0.5%). The plates were subsequently rinsed five times with acetic acid (1%) and allowed to air dry. The resulting purple residue was dissolved in Tris base (200 μl, 10 mM), and optical densities read on a ELx808 BioTek microplate reader. IC_50_ values were determined using the GraphPad Prism software.

### In Vitro PARP assay

The Trevigen HT Universal Colorimetric PARP assay kit with histone-coated strip well was used as per protocol provided by the vendor. Briefly, 50 μl per well of 1X PARP buffer was added to the strip well to rehydrate the histones and the plate was subsequently incubated at room temperature for 30 min. The solution was aspirated and replaced with a dose range of EG22 or ANI (10^−6^ to 100 μM) in triplicate. PARP enzyme (0.5 Unit/well) and a PARP cocktail were added to the appropriate wells containing the inhibitor. A negative control was prepared without PARP to determine the background absorbance, and a positive control without the inhibitor for a 100% reference point. After a 60-min incubation time, the strip wells were washed twice with 1X PBS + 0.1% Triton X-100 (200 μl/well) followed by 2 washes with 1X PBS. Some diluted Strep-HRP was then added after the washing and incubated for 60 min. Finally, a pre-warmed TACS-Sapphire colorimetric substrate was added to each well, in the dark, for 15 min at room temperature, after which the reactions were stopped by adding 0.2 M HCl. Optical densities at 450 nm were recorded on ELx808 Biotek microplate reader. The results were analyzed using GraphPad Prism software to derive a dose-response curve and the IC_50_ values. The PARP assay was performed twice, in triplicate.

### Alkaline comet assay for DNA damage quantification

Cells were plated in 6-well plates (Corning Inc.) at 200,000 cells/well in 2 mL medium/well. They were allowed to attach for 24 h and then treated with a wide range of drug concentrations (0, 6.25, 12.5, 25, 50. and 100 μM). The cells were exposed to the drugs (EG22, TMZ, and ANI + TMZ) for 2 h, harvested with trypsin EDTA, centrifuged and subsequently resuspended twice in PBS. The cell suspensions were mixed in low melting point agarose (0.75% in PBS) at >37 °C in a 1:10 dilution. The gels were cast on GelBond Film (Lonza, Switzerland) using gel casting chambers and allowed to solidify before being placed into a lysis buffer [2.5 M NaCl, 0.1 M tetrasodium EDTA, 10 mM Tris-base, 1% (*w*/*v*) N-lauryl sarcosine, 10% (*v*/v) DMSO, and 1% (*v*/v) triton X-100, pH 10.0]. They were subsequently kept at 4 °C overnight, rinsed with distilled water and immersed in a second lysis buffer [2.5 M NaCl, 0.1 M tetrasodium EDTA, and 10 mM Tris-base, pH 10.0] for 60 min at 37 °C after which they were gently rinsed with distilled water, incubated in alkaline electrophoresis buffer [0.3 M sodium hydroxide, 0.1 M tetrasodium EDTA, 7 mM 8-hydroxyquinoline, 0.2% (*v*/v) DMSO, pH 13.0] for 30 min at room temperature, and electrophoresed at 20 V, 400 mA for 20 min. Thereafter, they were gently rinsed with distilled water and placed in 10 M ammonium acetate for 30 min. Finally, the gels were soaked in 100% ethanol for 2 h, dried overnight, and stained with SYBR Gold (1:10,000 dilution of stock) (Molecular Probes, Eugene, OR) for 20 to 60 min. Comets were visualized at 400X magnification and DNA damage was quantified using Comet Assay IV software to calculate tail moments.

### Live cell Confocal microscopy

The V79 cell line was plated at 60–70% confluence in petri dishes, allowed to adhere overnight, and treated with 25 μM EG22, ANI and ZSM02 for 2 h. After treatment, cells were washed with PBS, a drop of DAPI (NucBlue® Live ReadyProbes® Reagent, ThermoFicher Scientific) was added and 3-D images were taken with the appropriate filter. Only the image corresponding to the equatorial plan of the cells was used to visualize cellular distribution.

### Kinetics of the hydrolysis of EG22 and ZSM02

The rate of hydrolysis of EG22 under physiological conditions was measured using a Spectra Max Gemini plate reader. The compound was dissolved in a minimum volume of DMSO and diluted with DMEM supplemented with 10% FBS. The solution was incubated in a 96-well plate at 37 °C in the ELISA reader and readings were taken over a period of 1 h. The excitation wavelength was 444 nm and emission 538 nm [27, 28]. The half-life was estimated from the formation of ANI using first order kinetics, one-phase exponential decay. (GraphPad software, Inc., San Diego, CA).

The stability of ZSM02 under physiological conditions was studied by HPLC, Agilent technologies. The compound was dissolved in minimum volume of DMSO and diluted with DMEM supplemented with 10% FBS. The solution was incubated at 37 °C and 100uL was collected at various time points: 0 min, 2 h, 4 h, 6 h, 12 h, 24 h, 48 h, 60 h, and 72 h. The drug was extracted from the media with 100 μL of methanol, centrifuged at 13,000 rpm for 1 min, after which the supernatant was collected and evaporated. The extraction was performed three times and after being dried in vacuo overnight, the resulting extract was reconstituted in 100 μL of methanol for HPLC analysis using a 150 mm × 4.6 mm ODS-3 (C18 column, 5 μm pore size) (Canadian Life Science). The absorbance was detected at 460 nm and the half-life estimated from the formation of ANI using first order kinetics analysis.

### Statistical analysis

Data were analyzed with Student’s two-tailed t-test or one-way ANOVA, using GraphPad Prism 5.0 software (GraphPad Prism, San Diego, CA). *P* < 0.05 was defined as statistically significant.

## Results

### Chemistry

**Fig. 4:**
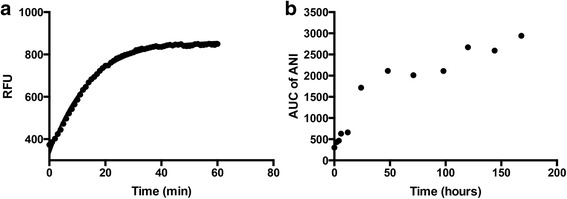
Hydrolysis of EG22 and ZSM02 in serum-containing medium. **a** The solution of EG22 was kept at 37 °C in the fluorescence reader and an intensity curve automatically generated at the maximum emission wavelength corresponding to ANI (538 nm) (t_1/2_ = 9.76 min); **b** ZSM02 was dissolved in a minimum volume of DMSO and diluted with DMEM supplemented with 10% FBS. The solution was incubated at 37 °C and 100 μL aliquots were analyzed by HPLC as described in Material and Methods. ZSM02 was slowly converted to ANI with t_1/2_ greater than 24 h

The proof-of-principle of the approach was first achieved by the synthesis of EG22, which proceeded according to Fig. 3. Using a known PARP inhibitor containing an aromatic amino group, 4-amino-1,8-naphtalimide (ANI), we designed EG22 to carry a 1,2,3-triazene moiety, which upon hydrolysis would regenerate ANI intact, while concomitantly releasing the DNA alkylating methyl diazonium ion, the latter species being identical to the one released by the clinical drug TMZ [13, 14]. EG22 was synthesized by diazotizing the amino group with sodium nitrite and adding methylamine under basic conditions. It was used as our first prototype to study the dual targeting of PARP and DNA with a single molecule in tumour cells. While EG22 was a useful probe for the combi-targeting of PARP and DNA, it was hydrolyzed too rapidly under physiological conditions (Fig. 4). Thus, we sought to delay its hydrolysis by acetylating its N3 nitrogen in pyridine cooled with liquid nitrogen prior. The unequivocal characterization of the resulting compound (ZSM02) by isotope labeling and heteronuclear NMR (^13^C, ^15^N) are reported elsewhere [25]. The structure was also confirmed by mass spectrometry, with a molecular ion at 296, consistent with its molecular weight.

### Dual PARP-DNA targeting properties of EG22

**Fig. 5:**
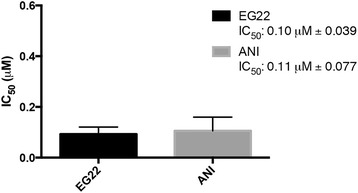
Enzymatic assay test for PARP inhibition by measuring the incorporation of biotinylated poly(ADP-ribose) onto histone proteins by the PARP enzyme. This allowed the determination of the IC_50_ value of our new PARP-DNA combi-molecule, EG22. The Trevigen HT Universal Colorimetric PARP assay kit with histone-coated strip wells was used and dose response curves analyzed with the Graphpad Prism software. The results showed that EG22 was capable of inducing a dose-dependent inhibition of PARP with IC_50_ = 0.1 μM

In order to verify whether EG22 could modulate its two targets (i.e. PARP and DNA), a PARP and a comet assay were performed to determine its ability to inhibit the function of PARP and to induce DNA damage, respectively. The known PARP inhibitor ANI could induce PARP inhibition in our assay with an IC_50_ of 0.11 μM, which is consistent with literature value (IC_50_: 0.16 μM) [29]. Our results showed that under the conditions of the assay, EG22 could induce a dose-dependent inhibition of PARP with an IC_50_ = 0.10 μM, which was in the same range as that of ANI (Fig. 5).

**Fig. 6:**
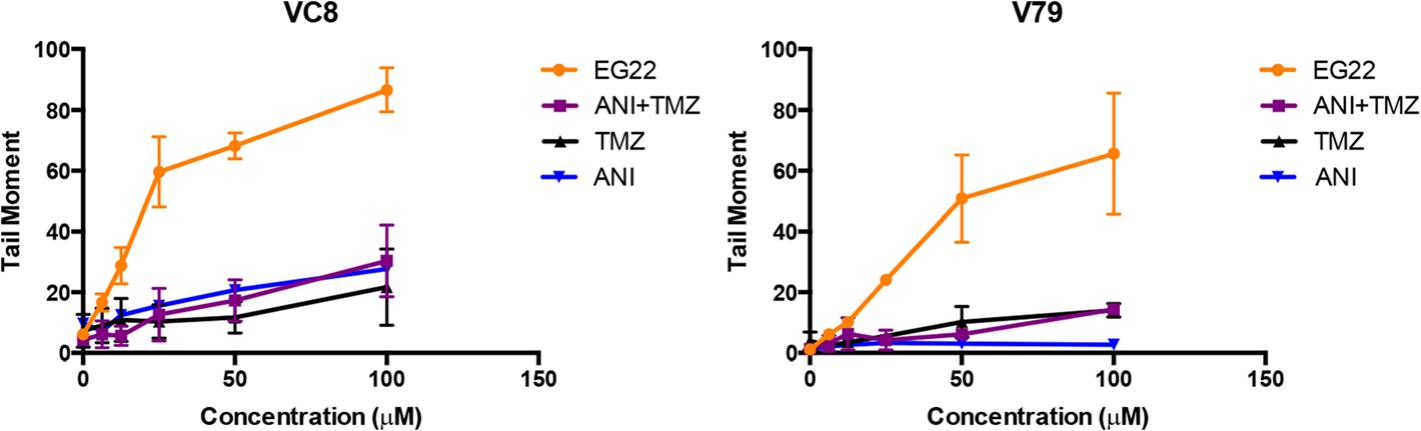
Anomalously strong DNA damaging potential of EG22 after a 2 h drug treatment as compared with temozolomide (TMZ) and ANI + TMZ in the Chinese lung cancer cell lines VC8 and V79. The cells were exposed to the drugs (EG22, TMZ, ANI and ANI + TMZ) for 2 h, and subsequently harvested with trypsin EDTA, centrifuged and resuspended twice in PBS. Comet assay was performed as per Materials and Methods. Comets were visualized at 400X magnification and DNA damage measured as tail moments using Comet Assay IV software

In order to determine whether EG22, a monomethyltriazene that like TMZ ultimately releases the methyl diazonium species, could induce DNA damage in tumour cells, we used the microelectrophoresis comet assay. The results showed that EG22 induced significantly higher levels of DNA damage than TMZ in a Chinese Hamster lung cancer BRCA2-mutant VC8 and proficient tumour cells V79 cells (Fig. 6). Interestingly, the levels of DNA damage induced by EG22 were significantly higher than those generated by the clinical drug TMZ. Since TMZ is the prodrug of the same alkylating species as EG22, the levels of the induced DNA damage appear anomalously high.

### BRCA1/2 response profile

**Fig. 7:**
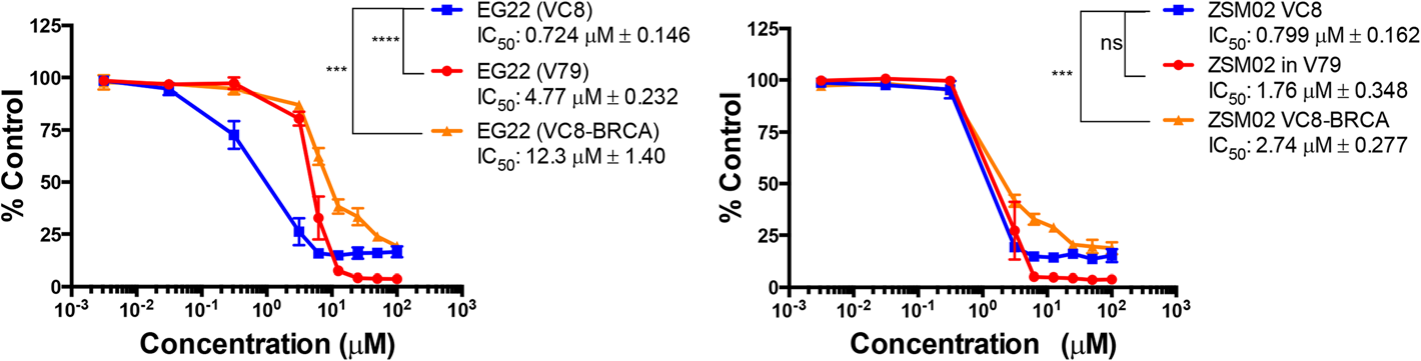
Dose response curve obtained from growth inhibition with EG22 in a panel of Chinese Hamster Lung cancer cell lines. V79 cells express wild type BRCA2.The VC8 cell line expresses mutant BRCA2, and VC8-BRCA is transfected with the wild type BRCA2 gene. EG22 was significantly more potent against the BCRA2 mutant cell line (*p* < 0.0001). ZSM02 did not show significant selectivity for VC8 when compared with V79. However, a 3-fold selectivity (*p* < 0.001) was apparent when compared with its isogenic VC8-BRCA counterpart. *** *p* < 0.001, ****p < 0.0001, ns. Not Significant

In order to verify whether the new combi-molecule could target BRCA1/2 mutants, we analyzed the potency of EG22 against the pair of Chinese Hamster lung cancer cell line: with V79, a BRCA1/2-proficient and the other VC8 BRCA1/2-mutant. The results showed that like the naked PARP inhibitor ANI, EG22 selectively killed the mutant forms (Fig. 7). Furthermore, in order to further ascertain the BCRA1/2 mutant selectivity of the approach, growth inhibition studies were performed in an isogenic context with VC8 cells (non-transfected) and VC8-BRCA (transfected with wild type BRCA2 gene). The results showed that EG22 was selectively more potent against the non-transfected VC8 cells (17-fold, *p* < 0.001) (Fig 7).

One of our goals was to verify whether the combi-molecule induced enhanced potency in the BRCA1/2 mutant. To this end, we compared the potency of EG22 with that of ANI, which is deprived of DNA alkylating functions. Importantly, the combi-targeted approach enhanced the potency of ANI in the BRCA2 mutants by 4-fold (*p* < 0.001, Table 1). It also enhanced ANI’s potency by 8-fold in the cells transfected with the wild type BRCA2 gene, which is an important advantage under conditions where resistance is associated with restoration of wild type BRCA1/2 [9, 10].

**Table 1:** Potency of EG22 on BRCA2-proficient and mutant Chinese lung cancer cell lines (IC_50_ μM)

	VC8	VC8-BRCA	V79
BRCA2 status	−	+	+
EG22	0.724 ± 0.146	12.3 ± 1.40	4.77 ± 0.232
ZSM02	0.799 ± 0.162	2.74 ± 0.277	1.76 ± 0.348
ANI	2.99 ± 0.567	>100	>100
TMZ	61.1± 12.3	>800	433.3 ± 55.9
ANI + TMZ	2.30 ± 0.552	83.5 ± 7.45	60.5 ± 10.6

### Relationship with MGMT status

To answer the question as to whether MGMT could affect the potency of EG22, we tested its potency in a panel of cells with known MGMT status, including an isogenic pair of human lung cancer cell line, A427 and A427 MGMT cell lines (Table 2). The results showed that MGMT expression did not affect the potency of EG22, indicating, that perhaps its ability to block PARP may enhance the cytotoxicity of DNA adducts other than O6-methylguanine in the cells. Unlike ANI + TMZ or TMZ alone, growth inhibition assays showed consistently strong potency of EG22 throughout the panel of MGMT positive cell lines. Indeed, EG22 was more than 13 to 47-fold more potent than the ANI + TMZ combination (*p* < 0.001), and 100- to 303-fold more potent than TMZ in the panel of cell lines. This shows that EG22 is capable of overcoming resistance to TMZ in the presence of MGMT.

**Table 2:** Potency of EG22 and ZSM02 on MGMT-proficient and deficient human tumour cells (IC_50_ μM)

	A427	A427 MGMT	A549	T98	DU145
MGMT status	−	+	+	+	+
EG22	3.8 ± 0.12	2.9 ±0.21	1.6 ±0.16	1.7 ± 0.16	6.0 ±0.55
ZSM02	2.9 ± 0.49	3.7 ± 0.23	2.0 ± 0.50	4.3 ± 1.6	4.7 ± 0.11
ANI	>100	>100	>100	>100	>100
TMZ	34 ± 4.0	305 ± 11	337 ± 28	516 ± 72	598 ± 59
ANI + TMZ	5.0 ± 0.82	41 ± 1.1	47 ± 11	80 ± 10	78 ± 4.6

### Potency of the combi-molecular approach in comparison with 2-drug combinations

**Fig. 8:**
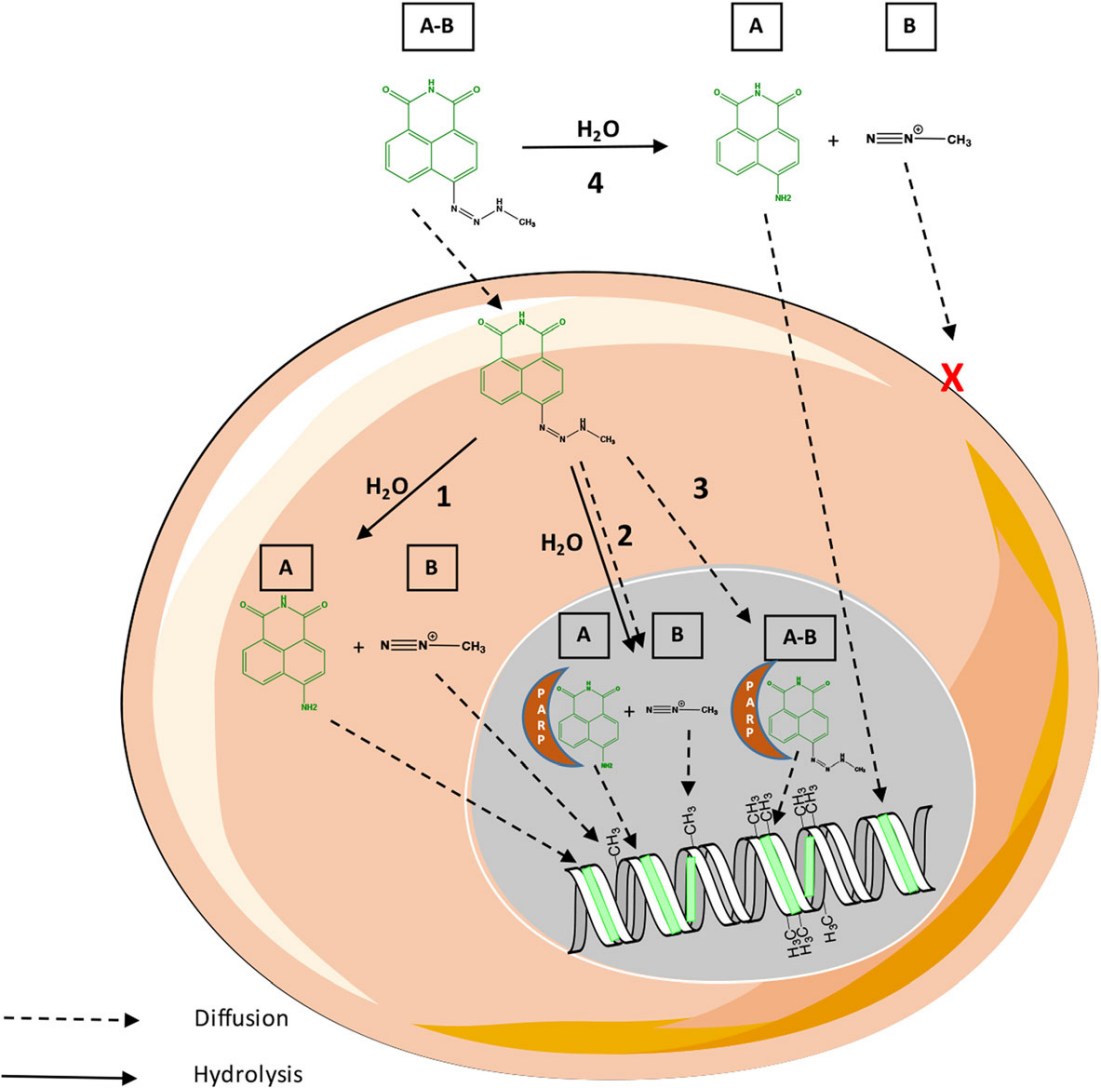
Proposed pathways for the hydrolysis of EG22 and its dual PARP-DNA targeting property. Solid arrows describe hydrolysis and dotted arrows diffusion. EG22 may diffuse in its intact form through the cell membrane to subsequently hydrolyze in the cytoplasm, release ANI and the methyl diazonium species. ANI may then in turn diffuse into the nucleus and either intercalate into the DNA or inhibit PARP. EG22 may also diffuse in its intact form toward the nucleus, intercalate into DNA prior to being converted to ANI and the methyl diazonium species

**Fig. 9:**
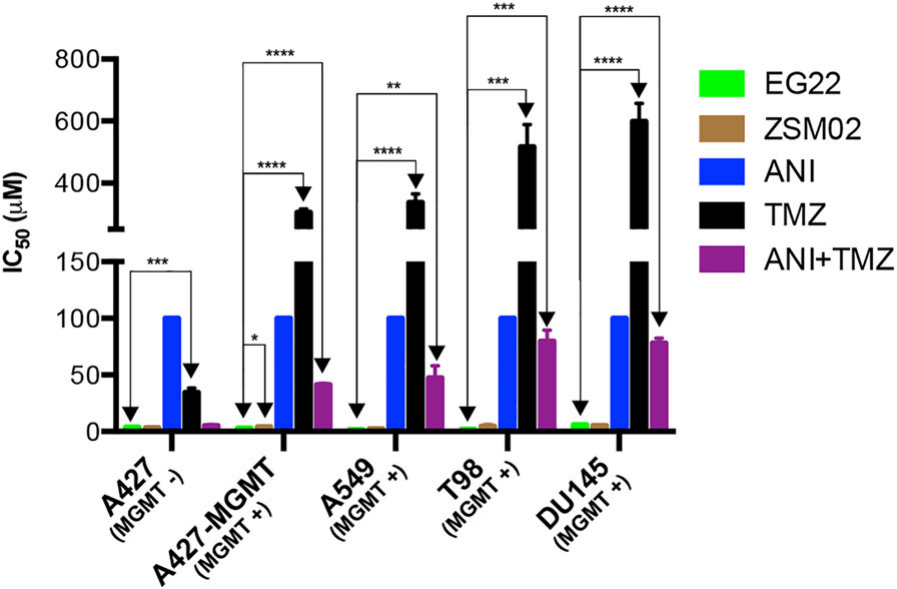
Growth inhibition by EG22 and ZSM02 in a cell panel with varied levels of MGMT. They show similar growth inhibition profile with an increased potency when compared to temozolomide (TMZ), ANI and ANI + TMZ, indicating that their potency is independent of the MGMT status of the cells

Importantly, the growth inhibitory potency of EG22 was 3-fold greater than that of the combination of ANI + TMZ against VC8 (mutant form), 13-fold in V79 (wild type) and 7-fold in VC8-BRCA, leading to an evidence of the ability of the combi-targeting approach to illustrate the principle underlying “the whole being greater than the sum of the part” (Fig. 8). The marked superiority of EG22 when compared with ANI + TMZ was further confirmed in a panel of established prostate, brain, and lung cancer cell lines (Fig. 9).

### Subcellular localization and mechanism of action

**Fig. 10:**
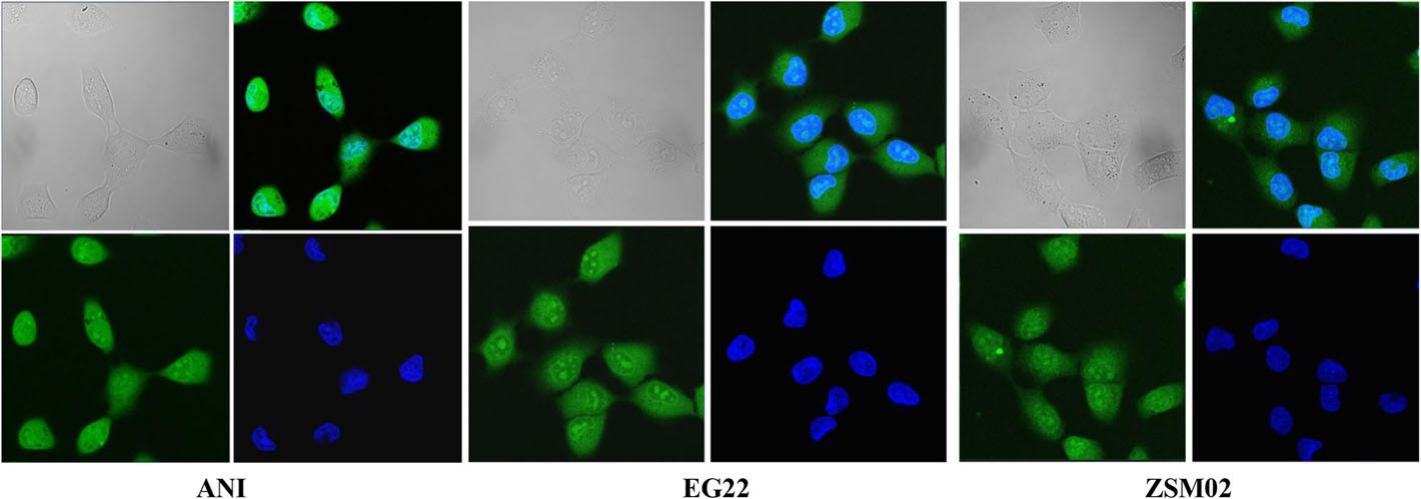
Subcellular distribution of ANI, EG22 and ZSM02 after a 2 h exposure. Following drug treatment, cells were washed with PBS, a drop of DAPI (NucBlue® Live ReadyProbes® Reagent, ThermoFicher Scientific) was added and 3-D images were taken with the appropriate filter. Nuclear localization of the drugs was confirmed by DAPI counterstaining

As shown earlier, the hydrolysis of EG22 leads to the release of ANI, an agent that fluoresces in the green and is also known to be able to intercalate into DNA [30]. Thus, its subcellular distribution was analyzed by fluorescence microscopy. Interestingly, as shown in Fig. 10, the green fluorescence was primarily localized in the nucleus, which is consistent with the fact that ANI can intercalate into DNA. This allowed us to propose a mechanism whereby, as depicted in Fig. 8, the intact molecule may be primarily localized in the nucleus where it generates ANI and its alkylating species in the nucleus, an event that may account for its ability to induce anomalously high levels of DNA lesions when compared with TMZ.

### Stabilization of EG22 and growth inhibitory profile of the resulting combi-molecule

Although EG22 has been shown to generate anomalously high levels of DNA damage as compared with TMZ, its rate of hydrolysis was considered to be too rapid under physiological conditions (t_1/2_ = 9.76 min) (Fig. 4). Therefore, we sought to stabilize it by acetylating the N3 of the triazene chain. As mentioned earlier, the stable form of EG22, known as ZSM02, has been synthesized and then analyzed by ^1^H, ^13^C, ^15^N NMR and mass spectrometry. Detailed NMR characterization of ZSM02 was reported elsewhere [25]. The potency profile of ZSM02 was studied in comparison with EG22. Although, it did not show selectivity for BRCA1/2 cells when its growth inhibitory potency was compared in the VC8/V79 pair of cell lines, in an isogenic context where VC8 is compared with its BRCA wild type transfectant, ZSM02 showed 3-fold selectivity for the mutant. Importantly, its potency in a panel of MGMT positive and MGMT negative cell lines paralleled that of EG22 (Fig. 7).

The kinetics of degradation of the stabilized molecule ZSM02 into ANI and methyldiazonium ion was studied and showed a slow release of the active species with a half-life greater than 24 h (Fig. 4) as opposed to the fast decomposition of the prototype molecule EG22. Thus, we have successfully stabilized EG22 by forming ZSM02.

## Discussion

The clinical potency of TMZ is significantly affected by the expression of several DNA repair enzymes, mainly MGMT [19, 31]. Attempts to overcome resistance to TMZ led to several approaches including direct inhibition of MGMT, blockade of abasic sites, PARP inhibition etc. [23, 32–38]. Alternative targets to sensitize cells to TMZ such as the epidermal growth factor receptor (EGFR) have been investigated by our laboratory [11, 39–43]. This has led to a novel tumour targeting strategy termed “combi-targeting” according to which molecules designed to block the action of their target and to be hydrolyzed into one inhibitor of the target + another bioactive species should induce strong potency in tumours expressing the target. Combi-molecules designed according to the combi-targeting concept are termed the type I (i.e. they are capable of releasing an inhibitor of EGFR + a DNA damaging species upon hydrolysis) or type II (i.e. they do not require hydrolysis to exhibit their binary EGFR/DNA targeting potency) (Fig. 1) [11, 12, 39–47]. Here, we sought to apply the type I model to the design of molecules capable of inducing a tandem PARP inhibition and DNA damage. Thus, a molecule (EG22) was synthesized that contained a PARP targeting moiety (ANI) and a methyl triazene tail designed to be hydrolyzed to a methyl diazonium species targeted to DNA (Fig. 2).

EG22 was not only capable of inducing about 7-fold greater potency against VC8 when compared with its V79 counterpart, but also displayed significant selectivity toward BRCA1/2 deficiency in the isogenic VC8/VC8-BRCA pair of cell lines. Importantly, it was generally more potent than TMZ against both BRCA mutants and wild type cells and when tested against VC8 transfected and its non transfected counterpart, indicating that it is synthetic lethality selective. It is to be noted that its superior potency when compared with BRCA1/2 wild-type and mutated cells is an advantage in tumours that express BRCA1/2 heterogeneously.

A PARP assay was performed to determine the PARP inhibitory potency of EG22 and an alkaline comet assay to demonstrate its DNA damaging properties. The strong PARP inhibitory potency of EG22 is consistent with the type I model of combi-molecules (Fig. 1), which are designed to release an intact inhibitor of the target upon hydrolysis. The rapid conversion of EG22 to ANI in cell culture medium suggests that the latter may be a major contributor to the PARP activity of EG22. Through the alkaline comet assay, the second arm of EG22 shows a dose-dependent DNA damage in both VC8 and V79 after 2 h and the levels of damage were significantly higher than those induced by TMZ and ANI + TMZ. We believe that the anomalously high DNA damage observed may be due to the primary intercalation of EG22 as an intact structure in DNA [27], where it releases its DNA damaging species. This may lead to a localized release of the DNA damaging species and enhancement of DNA damage in the cells. As depicted in Fig. 8, we propose that EG22 can degrade in the extracellular compartment, leading to ANI (path 4), which is capable of diffusing into the cells whereas the methyl diazonium is too unstable to penetrate the cells. EG22 can also enter the cells as an intact structure (paths 1 and 2) and decompose therein into ANI and the methyl diazonium species that are capable of reaching the nucleus. EG22 may intercalate into the DNA and release both ANI and methyl diazonium in situ (path 3). In the nuclear compartment, both intact EG22 and ANI can bind to and inhibit PARP. The proposed paths for the hydrolysis of EG22 and diffusion of the resulting by-products that appear to concentrate the DNA-targeting and damaging species in the nucleus, are consistent with the observed nuclear localization of the green fluorescence associated with ANI and the anomalously strong DNA damaging potential of EG22.

EG22 is designed to induce the same types of DNA adducts as TMZ, which is inactive against tumours expressing MGMT [14, 20], the sole human enzyme capable of repairing the O6-methylguanine adduct. It should be noted that despite its significant cytotoxicity, O6-methylguanine only accounts for 7% of base adducts induced by TMZ. N7-methylguanine and N3-methyladenine account for 70 and 10% respectively. The latter type of adducts are repaired by the base excision repair machinery [18]. EG22 being designed to induce the same types of lesions as TMZ and able to inflict significantly high levels of DNA damage to the cells while being a potent PARP inhibitor, we sought to determine whether its potency could be superior to that of TMZ in MGMT-expressing cells. Indeed, its 100–300-fold stronger potency in the panel of cells suggests that it is acting by a different mechanism of action when compared with TMZ. Perhaps, tandem blockade of PARP and induction of DNA damage allow to bypass the MGMT-mediated resistance. The levels of potency of EG22 were consistently similar throughout the cell panel whether the cells were BCRA1/2 wild type or mutant and MGMT+ or MGMT-. The ability of a PARP inhibitor to potentiate TMZ in tumour cells has already been reported [23, 35, 36]. However, to our knowledge this is the first report of a small 1,2,3-triazene-containing type I combi-molecules (MW = 296) capable of behaving like a PARP inhibitor and a DNA alkylating agent and more importantly with growth inhibitory potency stronger than that of a combination of two agents: a PARP inhibitor and a DNA damaging agent of the same structural class. Further attempt to enhance the druggability of the approach led the stabilization of EG22 by acetylating its N3-position to give ZSM02, which slowly released ANI and exhibited strong potency against MGMT cells. It showed less BRCA2 mutant selectivity than EG22, which is perhaps due to its ability to induce sustained release of the DNA damaging species concomitantly with ANI. This mechanism may depress the repair capacity of the wild type cells, thereby reducing the difference in potency when compared with the mutant. Nevertheless, the strong potency of ZSM02 that parallels that of EG22 in the MGMT-expressing cell panel warrants further investigation. Further studies are ongoing to assess its ability to behave as a true masked form of EG22 and to demonstrate its efficacy in vivo.

## Conclusion

In summary, EG22, our combi-molecule targeting PARP and damaging DNA, is the first prototype combining a PARP inhibitor (i.e. ANI) with an N-methyl-1,2,3-triazene as in TMZ and with a MW < 300. It is the first combi-molecule capable of releasing an aromatic amine preferentially localized in the nucleus, as opposed to the perinuclear localization that is typical of aminoquinazolines derived from the hydrolysis of EGFR-targeted combi-molecules reported by our laboratory [47]. Its ability to penetrate the cells and perhaps the nucleus where it may intercalate into DNA leads to an in situ generation of the DNA damaging species. This may account for its ability to generate anomalously high levels of DNA damage. The current work features a new type of DNA damaging agent with enhanced potency against BRCA1/2 mutants and MGMT-proficient tumour cells. Furthermore, its potency against BRCA1/2 wild type expressing tumours warrants strong activity against tumors in the advanced stages where BRCA1/2 becomes largely heterogeneous. Also, the strong potency of the approach against MGMT-proficient tumour cells indicates that type I agents like EG22 may be developed as a potential alternative to TMZ in advanced tumours characterized by MGMT expression.

## Abbreviations

AIC: 5-aminoimidazole-4-carboxamide
ANI: 4-amino-1,8-naphthalimide
BRCA1/2: Breast Cancer gene 1 or 2
DMEM: Dulbecco’s Modified Eagle Medium
DNA: Deoxyribonucleic acid
EGFR: Epidermal Growth Factor Receptor
FBS: Fetal Bovine Serum
MGMT: O6-Methylguanine Methyltransferase
PARP: Poly(ADP-ribose) polymerase
PBS: Phosphate-Buffered Saline
SRB: Sulforhodamine B
TCA: Trichloroacetic Acid
TMZ: Temozolomide
